# Intelligent Modeling and Multi-Response Optimization of AWJC on Fiber Intermetallic Laminates through a Hybrid ANFIS-Salp Swarm Algorithm

**DOI:** 10.3390/ma15207216

**Published:** 2022-10-16

**Authors:** Mahalingam Siva Kumar, Devaraj Rajamani, Ahmed M. El-Sherbeeny, Esakki Balasubramanian, Krishnasamy Karthik, Hussein Mohamed Abdelmoneam Hussein, Antonello Astarita

**Affiliations:** 1 Centre for Autonomous System Research, Department of Mechanical Engineering, Vel Tech Rangarajan Dr. Sagunthala R&D Institute of Science and Technology, Chennai 600062, India; 2 Industrial Engineering Department, College of Engineering, King Saud University, P.O. Box 800, Riyadh 11421, Saudi Arabia; 3 Department of Mechanical Engineering, Faculty of Engineering and Technology, Future University in Egypt, New Cairo 11835, Egypt or; 4 Department of Mechanical Engineering, Faculty of Engineering, Helwan University, Cairo 11732, Egypt; 5 Department of Chemical, Materials, and Industrial Production Engineering, University of Naples Federico II, 80138 Naples, Italy

**Keywords:** abrasive waterjet cutting, fiber intermetallic laminates, intelligent modeling, optimization, ANFIS, salp swarm optimization

## Abstract

The attainment of intricate part profiles for composite laminates for end-use applications is one of the tedious tasks carried out through conventional machining processes. Therefore, the present work emphasized hybrid intelligent modeling and multi-response optimization of abrasive waterjet cutting (AWJC) of a novel fiber intermetallic laminate (FIL) fabricated through carbon/aramid fiber, reinforced with varying wt% of reduced graphene oxide (r-GO) filled epoxy resin and Nitinol shape memory alloy as the skin material. The AWJC experiments were performed by varying the wt% of r-GO (0, 1, and 2%), traverse speed (400, 500, and 600 mm/min), waterjet pressure (200, 250, and 300 MPa), and stand-off distance (2, 3, and 4 mm) as the input parameters, whereas kerf taper (*Kt*) and surface roughness (*Ra*) were considered as the quality responses. A hybrid approach of a parametric optimized adaptive neuro-fuzzy inference system (ANFIS) was adopted through three different metaheuristic algorithms such as particle swarm optimization, moth flame optimization, and dragonfly optimization. The prediction efficiency of the ANFIS network has been found to be significantly improved through the moth flame optimization algorithms in terms of minimized prediction errors, such as mean absolute percentage error and root mean square error. Further, multi-response optimization has been performed for optimized ANFIS response models through the salp swarm optimization (SSO) algorithm to identify the optimal AWJC parameters. The optimal set of parameters, such as 1.004 wt% of r-GO, 600 mm/min of traverse speed, 214 MPa of waterjet pressure, and 4 mm of stand-off distance, were obtained for improved quality characteristics. Moreover, the confirmation experiment results show that an average prediction error of 3.38% for Kt and 3.77% for Ra, respectively, were obtained for SSO, which demonstrates the prediction capability of the proposed optimization algorithm.

## 1. Introduction

In recent times, composite materials are found to be an excellent alternative for structural, mechanical, and automotive applications instead of commercial materials such as metals and plastics because of the versatility in their properties, such as relative stiffness, high-strength-to-weight ratio, durability, rust, and corrosive resistance [1,2]. Fiber metallic laminates (FMLs) are a type of composite material that are created by fusing thin metal sheets as the skin material with fiber-reinforced composite materials, which provides a combined benefit of both metal and fiber composites [3,4]. Despite the potential advantages, producing near-net-shape end-use FMLs is still laborious because the manufacturing capabilities are infeasible and require the use of secondary manufacturing techniques such as cutting, drilling, milling, etc., to produce the necessary components for structural assembly [5].

The FMLs are layered composite materials, therefore, conventional machining of these materials is extremely difficult due to anisotropic and non-homogeneous structure, which leads to uneven thermal distribution, delamination in the matrix, rapid tool wear, etc. [6]. Therefore, in order to prevent damage to the surface and subsurface conditions of FMLs, unconventional machining processes, especially abrasive waterjet cutting (AWJC), are now preferred for processing these types of materials. AWJC is a mechanical energy-based manufacturing technique in which the material removal is performed by means of erosion due to the high pressurized abrasive slurry mixed waterjet impinged from a high-precision micro focusing tube at high velocity. Due to the absence of thermal effect and rapid cutting nature, AWJC is mostly preferred for processing a variety of materials including metals, ceramics and composites to achieve complex cutting geometries for stringent design requirements [7]. Despite the potential advantages, the non-linear and complex cutting mechanisms make it difficult to achieve attractive cutting features in AWJC parts, such as optimizing substrate material removal, fine-tuning kerf characteristics, and enhancing surface quality. It is caused by the presence of numerous process-related control factors, including water, abrasives, cutting, mixing, and acceleration parameters. Therefore, accurate mapping between output response characteristics and input process variables is essential for better comprehension, parametric analysis, process simulation, and machining process optimization.

Recently, numerous researchers have experimentally investigated the AWJC process in order to improve the quality of the processed components, especially for layered composite materials. Using the Taguchi methodology, Kalirasu et al. [8] investigated the effects of significant AWJC parameters on the kerf taper and surface roughness of a glass/coconut fiber-based hybrid composite. The investigation’s findings indicate that the factor most strongly impacting the chosen response characteristics is abrasive particle size. An experimental investigation was conducted by Hutyrová et al. [9] to investigate the surface topography of AWJ-machined wood plastic composites. According to their findings, AWJC can be utilized to effectively machine plastic composites without melting the matrix materials. For the assessment of the AWJC performance of jute/polyester composites, Kalirasu et al. [10] used an analytical method called response surface methodology and multi-objective optimization on the basis of ratio analysis (RSM-MOORA). They investigated how stand-off distance, cutting speed, and jet pressure affected the kerf taper and surface roughness of machined surfaces. The findings show that the proposed method can be used effectively for fiber-reinforced composites up to a maximum thickness of 3 mm. Using the response surface approach, K. Balamurugan et al. [11] investigated the AWJC characteristics of a composite made of lanthanum phosphate and yttria. They considered the impact of kerf taper, surface quality, and material removal rate as well as cutting speed, stand-off distance, and water pressure as input factors. Their findings showed that cutting speed has a negative impact on surface quality whereas water pressure has a positive impact on kerf taper and material removal rate. To examine the surface and kerf characteristics of AWJC of stacked Ti and carbon fibre reinforced plastics (CFRP) FMLs, Pahuja et al. [12] carried out experimental and statistical analyses. They asserted that the top skin is where micro buckling and fracture start to cause the erosion mechanism of FMLs. Additionally, the designs of the metal skin and polymer composite have a big impact on the FML interface failures. The effect of AWJC factors on the kerf quality of machined Ti/CFRP composite stacks has been analytically investigated by Ramulu [13]. They created an empirical model to anticipate the depth of penetration and material removal mechanism caused by waterjet pressure, and they discovered that the model can be successfully utilized to control AWJC parameters to produce cutting zones with no defects. On the qualitative traits of Ti/CFRP stacked composite laminates, a drilling operation through AWJ machining was carried out by Alberdi et al. [14]. Their findings showed that the layout of stacks, water pressure, and traverse speed of the focusing tube have a significant impact on the taper of drilling and surface quality.

Recently, few researchers have utilized intelligent modeling such as fuzzy logic, artificial neural network (ANN), adaptive neuro-fuzzy inference system (ANFIS), and metaheuristic optimization algorithms to obtain the optimal cutting conditions of the AWJC process [15,16,17,18,19]. However, none of the researchers have hybridized the metaheuristic algorithms to optimize ANFIS parameters to correlate the relationship between the input and response characteristics of the AWJC process, especially for fiber metallic laminates made of nickel titanium superalloy (Nitinol) as the skin material. A titanium dioxide-based protective surface layer protects nitinol from corrosion. Nitinol’s biocompatibility and corrosion resistance are excellent when properly processed, and it provides the flexibility and strength required for many applications. When Nitinol is combined with carbon/aramid-based epoxy prepregs, it results in excellent structural stability with high impact strength.

The emphasis of the current work is on the AWJC of a novel fiber intermetallic laminate (FIL) made of sheets of Nitinol shape memory alloy and reduced graphene oxide (r-GO) filled epoxy prepregs embedded with carbon and aramid fibers, which are alternately interlaced. Among the available fabrication techniques, the vacuum resin infusion process has been utilized for fabricating the FILs due to their significant advantages such as high consistency, repeatability, minimized styrene emissions and a cleaner process than traditional FRP processing. The kerf taper and surface roughness were considered as independent response characteristics for the investigation. The AWJC experiments were carried out by varying processing parameters such as traverse speed, waterjet pressure, and stand-off distance as the independent variables with the different wt% of r-GO fillers in the FILs. Hybrid intelligent modeling of a parametric tuned ANFIS through metaheuristic algorithms was performed for correlating the relationship between the input parameters and output responses. Finally, the optimized ANFIS network was optimized through the salp swarm optimization (SSO) algorithm for obtaining optimal AWJC parameters.

## 2. Methodologies

**Figure 1:**
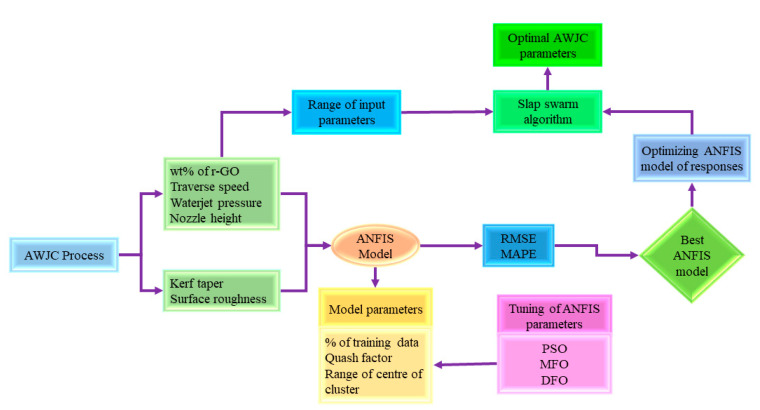
The flowchart of proposed modeling and optimization methodology.

The proposed work aims to adopt a hybrid approach of metaheuristic algorithm tuned ANFIS intelligent modeling of AWJC parameters and multi-response optimization through the salp swarm optimization algorithm. The flowchart shown in Figure 1 indicates the proposed methodology to achieve the modeling and optimization of the AWJC process. In the first phase, the ANFIS prediction model was proposed in order to correlate the AWJC parameters and output responses. The ANFIS model parameters were further optimized through metaheuristic algorithms such as particle swarm optimization (PSO), moth flame optimization (MFO), and dragonfly optimization (DFO), and the best algorithm was selected based on the obtained prediction errors such as root-mean-square error (RMSE) and mean absolute percentage error (MAPE). In the second stage, the ANFIS model was trained based on the optimal parameters and the prediction performance was evaluated with the actual response values. In the final stage, the optimized ANFIS network was considered as the objective function for multi-response optimization and the salp swarm optimization algorithm was conceived for obtaining the optimal AWJC parameters. The proposed methodologies such as response surface methodology (RSM), ANFIS, and the SSO algorithm were discussed in subsequent sections.

### 2.1. Response Surface Methodology

In order to develop empirical models without sacrificing their prediction ability, advanced manufacturing processes are adopting novel designs of experimental approaches to reduce the number of experimental trials. Since the AWJC process involves several uncertain processing conditions that significantly influence the quality features of the processed components, a competent experiment design approach, namely the Box–Behnken design (BBD) of response surface methodology, has been used for designing the viable number of experiments. In this present study, four parameters such as the addition of r-GO, traverse speed, waterjet pressure, and stand-off distance of each of the three levels have been considered for conducting the experiments with five replications for predicting the output responses such as kerf taper and surface roughness. The second order empirical models can be developed through the proposed BBD approach, which has been expressed as follows (Equation (1)):

(1) $$R=C_{0}+\sum_{i=1}^{k}C_{i}x_{i}+\sum_{i}\sum_{j}C_{ij}x_{i}x_{j}+\sum_{i=1}^{k}C_{ii}x_{i}^{2}+\omega$$

where ω signifies statistical distribution error, $C_{i}$, $C_{ij}$, and $C_{ii}$ indicates the linear, interaction, and quadratic coefficients of the parameters, respectively, R indicates the response value and k indicates the number of dependent variables.

### 2.2. Adaptive Neuro-Fuzzy Inference System (ANFIS)

ANFIS is a hybrid machine learning intelligence model, which comprises the advantages of both fuzzy logic and neural network for precise mapping of the correlation that exists between the input and output parameters of complex real-world problems [20]. In ANFIS, the Takagi–Sugeno fuzzy inference system has been optimized by the highly interconnected neural network models, which enables the network to be more reliable for predicting the relationship between uncertain variables associated with any system. The general ANFIS network architecture consists of five major steps, as follows: input fuzzification, implication, normalization, defuzzification, and output layers. The detailed step-by-step explanation of how the ANFIS network has been effectively utilized for intelligent modeling of the advanced manufacturing process is explained by the previous works [21,22].

### 2.3. Salp Swarm Optimization (SSO) Algorithm

**Figure 2:**
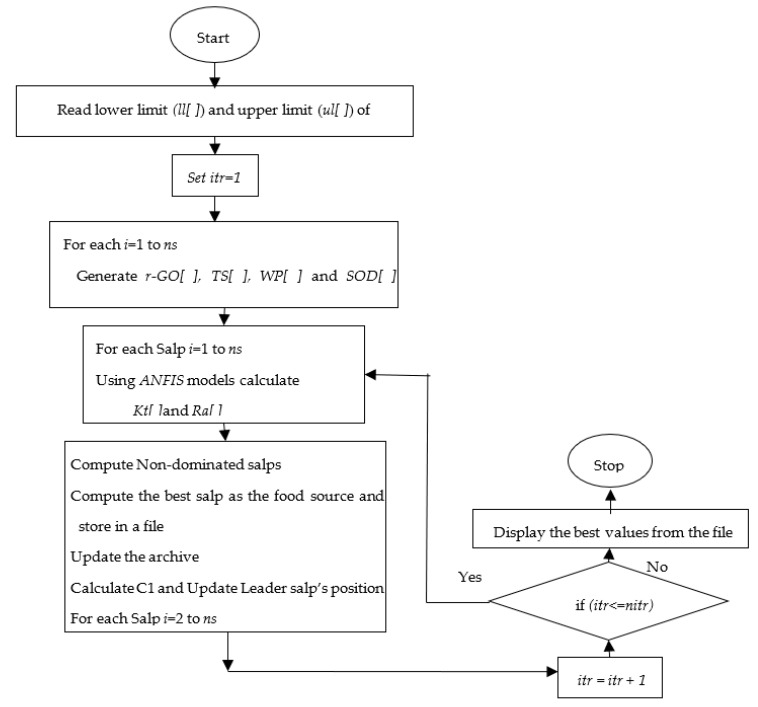
Implementation flowchart for salp swarm optimization algorithm.

Salp swarm optimization (SSO) is a novel swarm-based metaheuristic algorithm that mimics the navigating and foraging mechanism of salps in the oceans [23]. In the SSO algorithm, both leader and follower salps form the salp chain. The salp at the top of the salp chain is the leader, and the other salps are regarded as the followers. Half of the salps are chosen as leaders in order to increase the population diversity of the algorithm and the capacity to exit the local optima. The implementation flowchart of the proposed SSO algorithm for solving this engineering problem has been depicted in Figure 2. The step-by-step procedure of the SSO algorithm for obtaining optimal AWJC parameters is as follows:

*Step 1*: Initialization of salp population

A set of parameters $\left(P_{ij}\right)$ such as wt% of r-GO, TS, WP, and SOD are generated within lower bound and upper bound values as per their selected levels using the following relation (Equation (2)).

(2) $$P_{ij}=lb_{j}+rand*\left(ub_{j}-lb_{j}\right)$$

where $lb_{j}$ and $ub_{j}$ are the lower and upper bound value of $j^{th}$ parameter, and a *rand*-random value between 0 and 1.

*Step 2*: Evaluation of fitness of each Salp

The fitness values of each salp, i.e., responses such as *Kt* and *Ra*, are calculated using the optimized ANFIS Model. The non-dominated salps, based on their dual objectives, are computed and the best salp, based on the crowding distance, is assumed as food source $\left(F_{1j}\right)$ and stored in a file. The archive size is updated within its maximum size.

*Step 3*: Update the leader position and the other salps’ position

The value of the squared exponential covariance variable (C1), which defined the position of the leader salp, is calculated using the following relation (Equation (3)).

(3) $$c_{1}=2e^{-{\left(\frac{4it}{\max\_it}\right)}^{2}}$$

where it is the current iteration number and max_it is the maximum iteration number or stopping criteria. The leader salp’s position is updated through the following relation (Equations (4) and (5)).

(4) $$if\;c_{3}<0.5\;P_{1j}=F_{1j}+c_{1}\left[\left(ub_{j}-lb_{j}\right)c_{2}+lb_{j}\right]$$

Else

(5) $$P_{1j}=F_{1j}-c_{1}\left[\left(ub_{j}-lb_{j}\right)c_{2}+lb_{j}\right]$$

End

where $c_{2}$ and $c_{3}$ are random values between 0 and 1, $lb_{j}$ and $ub_{j}$ are the lower and upper bound value of $j^{th}$ parameter, and $F_{1j}$ is the position of food source. Further, the position of follower salps is described as follows (Equation (6)):

(6) $$P_{1j}=\frac{1}{2}\left(P_{1j}+P_{\left(i-1\right)j}\right)$$

Finally, the updated position of salps is verified, whether they are within their bounds, i.e., $lb_{j}$ and $ub_{j}$.

*Step 4*: The initial position of salps is updated with the new updated position using the entire replacement strategy.
*Step 5*: Starting from step 2 to step 4, these steps are repeated until the stopping criteria.
*Step 6*: The dual objective solutions from the archive are converted into single objective solutions by implementing Deng’s method. The order preference of the solution is made based on how high the value is in the overall performance index.
*Step 7*: The optimum process parameter (wt% of r-GO, TS, WP, and SOD) and its responses (*Kt* and *Ra*) are displayed based on the higher overall performance index value.

## 3. Experimental Procedure and Measurements

The FILs are fabricated through a vacuum assisted resin infusion process by utilizing Nitinol shape memory alloy sheet as the skin material, bidirectional weaving patterned carbon and aramid fibers as the prepreg fiber materials, and reduced graphene oxide (r-GO), a derivative of graphene, which has been prepared through the modified hummer’s method and has been supplemented as nano filler in the matrix material. The r-GO nano filled epoxy matrix is supplied through a resin inlet port of the infusion process in order to fabricate FILs with alternatively stacked woven carbon and aramid fibers with Nitinol shape memory alloy foils (NiTi/[carbon/epoxy/aramid]8/NiTi) as a skin material. The final thickness of fabricated FILs is upheld to approximately 3.5 mm.

**Figure 3:**
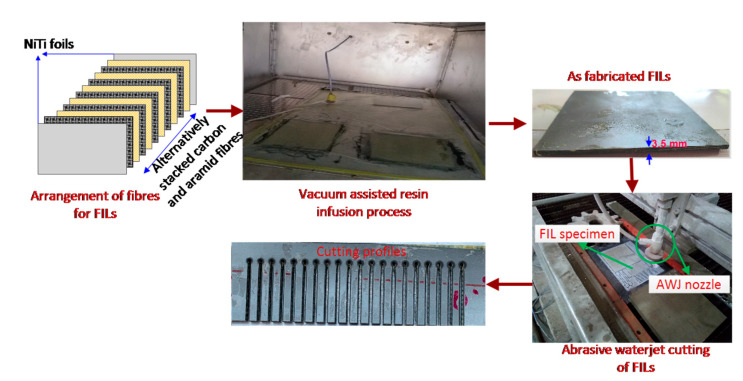
Development and AWJ cutting of fiber intermetallic laminates.

**Figure 4:**
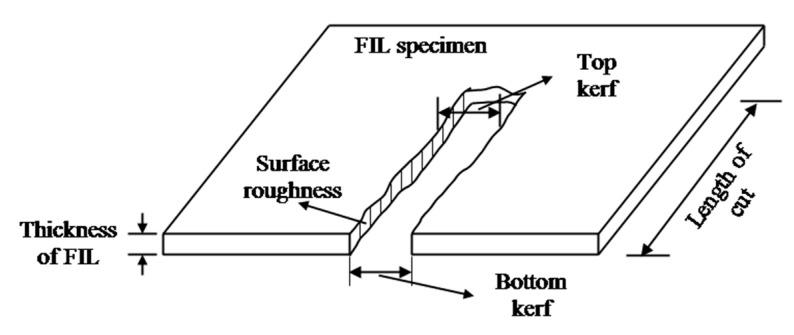
Schematic of measuring the quality characteristics such as *Ra* and *Kt*.

The cutting experiments were performed on three different categories of FILs fabricated with 0, 1, and 2 wt% of r-GO nano fillers through a high-precision cantilever type computer numerical controlled AWJC system (S3015, Waterjet Germany, Chennai, India). The AWJ machine is equipped with a high-pressurized intensifier pump of operating pressure ranging up to 450 MPa and a carbide focusing tube of 0.76 mm diameter to achieve efficient machining operations. The machining parameters, such as the abrasive flow rate of 200g/min, the garnet abrasive particle size of ~177µm (80 mesh), the orifice diameter of 0.276 mm and the jet impact angle of 90°, are constantly maintained throughout the experiments. As per the RSM-BBD experimental design strategy for four dependent variables with three levels such as r-GO addition of 0, 1, and 2 wt%, traverse speed of 400, 500, and 600 mm/min, waterjet pressure of 200, 250, and 300 MPa, and stand-off distance of 2, 3, and 4 mm, a total of 29 straight parallel slots were made in the different FILs with a cutting length of 50 mm for the investigation of *Kt* and *Ra*. The range of AWJC parameters was selected by performing extensive preliminary studies. The typical AWJC experimental setup and processed FIL specimens is depicted in Figure 3. The AWJC system consists of abundant process-related dependent variables, whereas the most significant variables such as TS, WP, and SOD, suggested by previous researchers, were selected for the present investigation. The kerf taper at jet entry and exit of the machined FILs were computed using a METZ 1395 (Metzer Instruments, Mathura, India) tabletop microscope and the surface quality at the kerf side surface was measured using a non-contact three-dimensional profilometer (Talysurf CCI Lite, Taylor & Hobson, Leicester, UK). The computational errors were minimized by measuring *Kt* and *Ra* thrice and their mean values was considered as responses. The schematic representation of measuring Ra and Kt has been shown in Figure 4. The response features obtained from the AWJC of FILs are further considered for statistical validation and optimization, as presented in Table 1.

## 4. Results and Discussion

### 4.1. Statistical Analysis of Developed Mathematical Models

**Figure 5:**
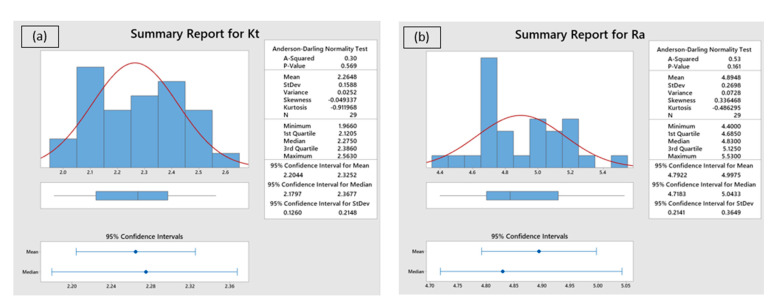
Statistical validation of response characteristics: (**a**) *Kt*, and (**b**) *Ra*.

The adequacy and statistical significance of the developed mathematical models and performed experimental investigations were assessed through multi-analysis of variance (ANOVA) and Anderson–Darling normality tests. The experimental work considered the most significant AWJC parameters that influence the quality features of processed components. Therefore, the influence of individual parameters on the selected responses should be assessed through a systematic analysis for improving the cut quality characteristics. The ANOVA results for selected response features such as *Kt* and *Ra* have been mentioned in Table 2 and Table 3. From the results, the coefficient of determination values for *Kt* and *Ra* is achieved at 99.1% and 99.2%, respectively. Moreover, the insignificant terms at a 95% confidence interval were eliminated from the regression modeling through the backward elimination procedure. The lack-of-fit values were also found to be significant within the selected range of processing parameters, which signifies the statistical significance of the conducted experiments. In addition, the statistical analysis (Anderson–Darling normality test) is performed to validate the obtained solutions at a 95% confidence level (Figure 5). The results of statistical analysis also revealed that the obtained solutions are statistically significant and are normally scattered against actual values, which confirms the accuracy of conducted AWJC experiments.

### 4.2. Intelligent Modeling of AWJC Quality Characteristics through Optimized ANFIS Network

Due to the uncertainty in non-traditional machining processes such as abrasive waterjet machining, the correlation that exists between the input parameters and the output characteristics is extremely non-linear. As a result, an efficient approach to modeling the relationship between the parameters is critical in order to improve the predictability of such processes. The correlation between the AWJC parameters such as traverse speed, waterjet pressure, and stand-off distance, as well as r-GO addition in FML on response features such as *Kt* and *Ra* was established in this work using the ANFIS intelligent modeling approach. To develop the proposed hybrid intelligent model, a customized code was written in the MATLAB 2021b™ environment. The proposed work utilized a sugeno-type sub-clustering “genfis2” ANFIS model for appropriate modeling of the inference system. Since the ANFIS network parameters, such as premise and consequent variables, are generally selected based on the choice of the user and/or a trial-and-error approach, this may lead to an inefficient model with a quantum of prediction errors. Therefore, an efficient tuning approach is necessitated to optimize these parameters to improve the prediction capability of the ANFIS network.

In the present work, three well-established metaheuristic algorithms namely PSO, MFO, and DFO have been efficiently utilized for tuning the ANFIS parameters in order to improve the learning capability by minimizing the prediction error. In general, the behavior of the subtractive clustering ANFIS model is influenced by the RADII, squelch factor, accept ratio, and rejection ratio. The RADII clustering center, which ranges from 0 to 1, represents the influencing ranges of each dependent and predictor variable. In several cases, it was discovered that a smaller cluster RADII is a better option for obtaining better prediction results. Therefore, a minimum range of RADII values between 0.13 and 0.5 were considered in the current work. The neighborhood cluster center was calculated by multiplying a chosen range of RADII values by the quash factor. Additionally, the volume of data required for effective training and performance testing of the FIS model is considered. The default values for the acceptance and rejection ratios are frequently 0.5 and 0.15, respectively. For each response, the RADII, squelch factor, number of training sessions, and data verification are additional factors that must be taken into consideration when building the best FIS model.

In the first step of ANFIS hybrid modeling, the control elements of the AWJC process, such as traverse speed, waterjet pressure, and stand-off distance with wt% of r-GO, are specified as input factors, whereas *Kt* and *Ra* are thought of as output factors. Furthermore, to increase the trained network’s accuracy while reducing prediction errors, FIS parameters such as cluster radius, quash factor, and proportion of data used to train the network are selected. Table 4 contains a list of the ANFIS variable levels considered for the network’s initial training with selected metaheuristic algorithms. The fuzzy rules are designed to cluster the chosen processing variables into a variety of values by combining two or more membership functions. Extensive membership functions must be generated in order to develop a rule-based correlation between the processing variables and response characteristics because there are numerous process parameters. As a result, this research utilizes the subtractive fuzzy clustering approach. Due to their smoothness and concise notation when estimating the responses, Gaussian-shaped membership functions (MFs) are preferred over a variety of other available membership functions (MFs). The key contributing parameters for PSO, MFO, and DFO for optimizing the ANFIS parameters have been mentioned in Table 5 and Table 6. These parameters, which are the ideal training parameters for the current investigation, were obtained through multiple iterations of the trial-and-error method.

**Figure 6:**
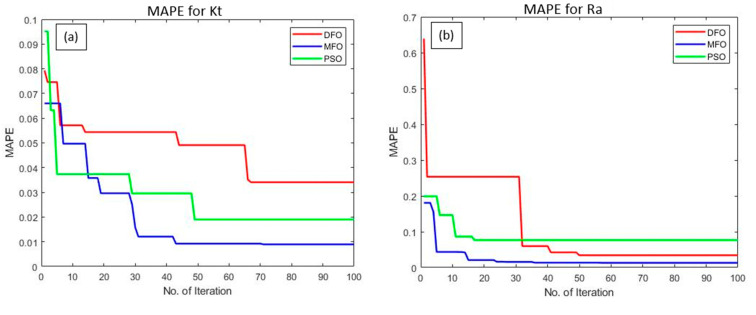

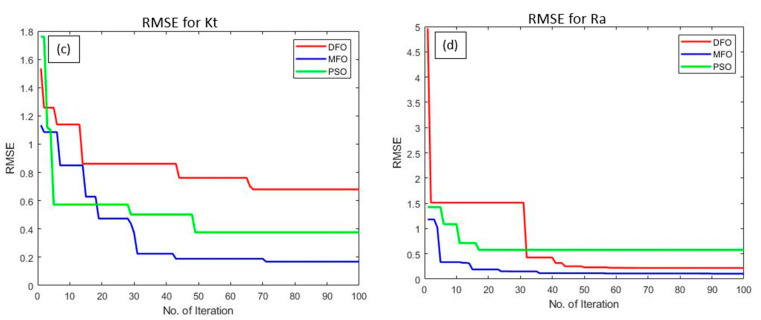
Convergence plots for: (**a**,**b**) MAPE, and (**c**,**d**) RMSE of *Kt* and *Ra*.

**Figure 7:**
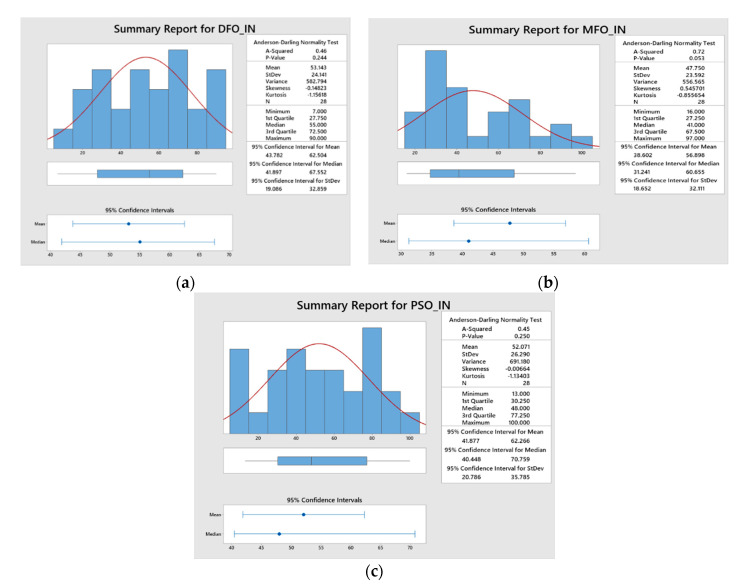
Statistical analysis for MAPE obtained from: (**a**) DFO, (**b**) MFO, and (**c**) PSO.

**Figure 8:**
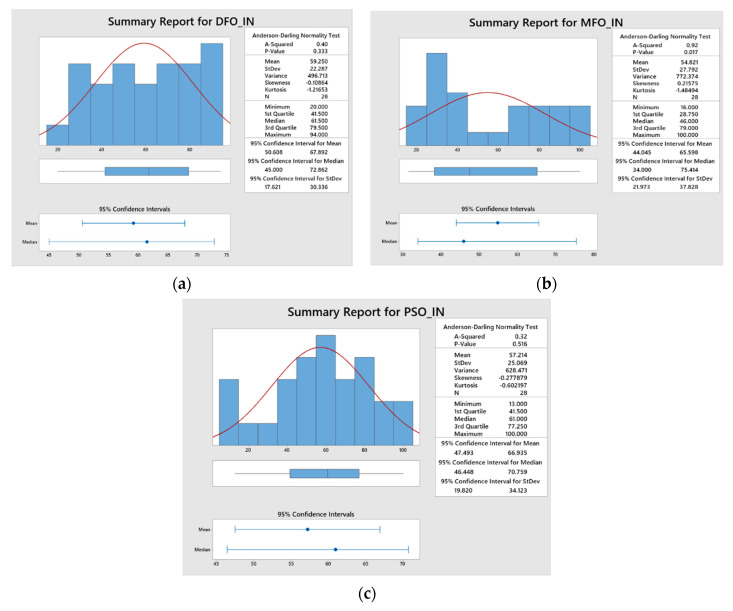
Statistical analysis for RMSE obtained from: (**a**) DFO, (**b**) MFO, and (**c**) PSO.

The proposed algorithms were executed several times for improving the prediction capability of the ANFIS network by minimizing the MAPE and RMSE during the training of the network. The algorithms were simultaneously executed for 100 iterations in order to obtain the optimal ANFIS parameters. The convergence plots of DFO, MFO, and PSO algorithms as shown in Figure 6a–d, which indicates the correlation between the number of iterations and their consequences on the ANFIS outputs such as MAPE and RMSE for the response features such as *Kt* and *Ra*. The convergence plots show that the MFO algorithm has converged effectively and produced minimized MAPE and RMSE values when compared with the other two optimization algorithms. In addition, the statistical analysis results (normal distribution of data) have also proved that the MFO algorithm is efficient in the training of the proposed ANFIS network parameters at a 95% confidence interval, with an improved Anderson squared value of 0.72 for MAPE and 0.92 for RMSE, respectively (Figure 7 and Figure 8).

**Figure 9:**
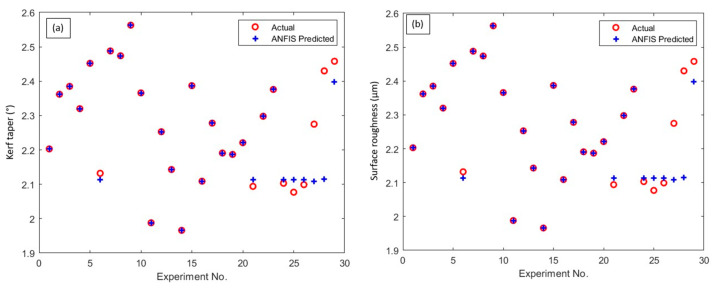
ANFIS predicted response values vs. experimental measured values: (**a**) *Kt*, and (**b**) *Ra*.

The optimal ANFIS parameters and their corresponding error values obtained for 30 epochs through the proposed optimization algorithms have been illustrated in Table 7. Among the available RMSE and MAPE values, MFO produced values are considered minimal for both the responses such as *Kt* and *Ra*. The optimized results obtained through MFO, 80% of the experimental data, i.e., 23 data, were considered for training the network, whereas the remaining six experimental data were taken to evaluate the prediction ability of the trained network. The objective of ANFIS intelligent modeling is to minimize the error between the training and checking data for each response. From the table, the training and checking errors obtained through the MFO algorithm for *Kt* is 0.0056% and 0.016%, and for Ra is 0.0088% and 0.0231%, respectively. The prediction error between the actual data and ANFIS predicted data has been graphically represented in Figure 9a,b. These findings suggest that the error values between the training and checking data are found to be minimal. As a result, this proposed trained ANFIS approach could be used to accurately predict AWJC parameters with fewer prediction errors.

### 4.3. Influence of Process Parameters on Quality Characteristics

The kinetic energy of the waterjet and its associated variables, as well as the thickness of the substrate materials, have a significant impact on the cutting quality in the AWJC process. The primary cause of an irregular kerf taper ratio, which lowers the calibers of machined components, is the energy depletion of the waterjet along the thickness of the substrate. To achieve the desired cutting quality with minimized taper, appropriate AWJC variables must be chosen and their effects on kerf taper must be investigated. Moreover, to determine how the machined components will interact with their adjacent components and various working environments, it is crucial to investigate the surface quality of abrasive waterjet machined materials, especially layered composites.

**Figure 10:**
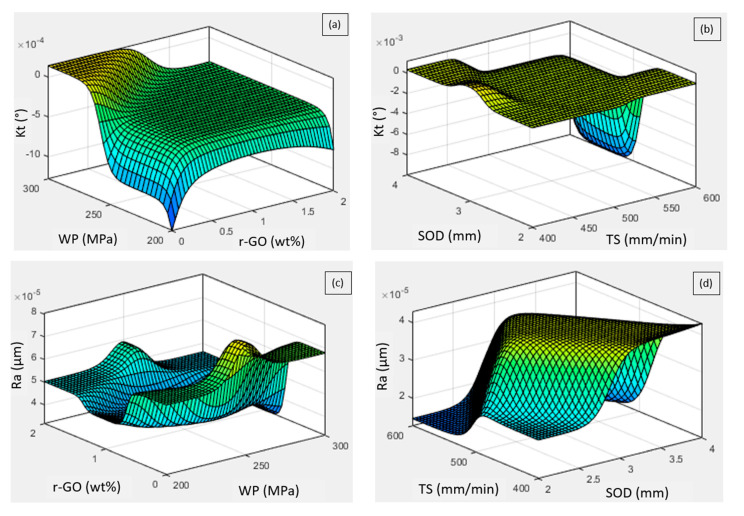
Influence of process parameters on the response characteristics: (**a**,**b**) Influence on *Kt*, and (**c**,**d**) Influence on *Ra*.

The influence of various AWJC parameters and the inclusion of r-GO with varying weight percentages on the quality characteristics of processed FILs at varying cutting conditions are described in the ANFIS surface plots (Figure 10a–d). The influence of waterjet pressure (WP) and wt% of r-GO on *Kt* is represented in the 3D surface plot (Figure 10a). The plot reveals that *Kt* linearly increases with an increase in WP from 200 bar to 300 bar, whereas a slight variation in *Kt* has been found when the addition of r-GO has been increased from 0 to 2 wt%. When the waterjet pressure is increased, the FILs may be impinged upon by the kinetic energy and flow turbulence of the water-jet stream, creating a wider slot. In addition, interparticle collision is increased by flow turbulence and water-jet expansion, which widens the kerf surface’s taper [12,24]. The consequences of traverse speed (TS) and stand-off distance (SOD) on *Kt* have been demonstrated in Figure 10b. The plot shows that *Kt* is linearly widening with an increase in SOD from 2 mm to 4 mm and TS from 400 mm/min to 600 mm/min. This might be because of a narrowing of the effective waterjet caused by higher SOD, which causes the waterjet to flow away from the focusing tube and contract into abrasive particles. As a result, the cutting and penetrating zones of abrasive particles in a waterjet are reduced in energy. The kerf on the bottom cut surface is consequently smaller than the kerf on the top cut surface. *Kt* was therefore found to be greater at higher SOD levels [25].

The combinatory effects of the AWJC process parameters on *Ra* have been represented by the ANFIS three-dimensional plots (Figure 10c,d). The influence of waterjet pressure and r-GO addition on surface quality has been presented in Figure 10c. It is perceived from the plot that *Ra* has been increased linearly with an increase in waterjet pressure, whereas the addition of r-GO nano fillers is found to be insignificant on surface quality. As the waterjet pressure increases, the energy fluctuation of the waterjet also intensifies. The high-pressure waterjet’s erosion of abrasive particles will make the cut surface more uneven, which results in a rough-cut surface [26]. A three-dimensional surface plot (Figure 10d) shows the effect of the stand-off distance and traverse speed on the *Ra* of processed FIL surfaces. As can be seen from the plot, the *Ra* is significantly increasing, with an increase in both the processing variables from their low to high levels. When the stand-off distance rises, the waterjet expands before impacting the substrate, exposing the substrate to more extrinsic drag from the surrounding environment. As a result, the kinetic energy of the jet is reduced, causing the jet diameter to expand, resulting in a rough-cut surface [25,27].

### 4.4. Multi-Response Optimization through Salp Swarm Algorithm

In this study, a hybrid approach of a parametric tuned ANFIS-salp swarm optimization algorithm has been used for the modeling and optimization of AWJC parameters during the processing of novel fiber intermetallic laminates. Based on the results of the proposed hybrid approaches, the following inferences were attained:

The present investigation aims to optimize the AWJC parameters in order to improve the cut quality characteristics such as kerf taper and surface quality. In general, the multi-response optimizations were performed in two ways: (i) the objective functions were combined into single objectives by assigning weight for each function to minimize the optimization complexity, and (ii) to obtain optimal results through pareto-optimal solutions of the deciding parameters [28]. In this study, the optimization was performed through a novel metaheuristic-based salp swarm optimization algorithm in order to obtain the non-dominated optimal solutions. The trained ANFIS outputs for each response have been considered as the objective functions for the multi-response optimization. The SSO algorithm was executed with the correlation of terms as indicated in Table 8. The implementation of SSO is simple and only needs a few parameters. Therefore, the algorithm was able to produce the best results possible with these minimal parameter settings.

**Figure 11:**
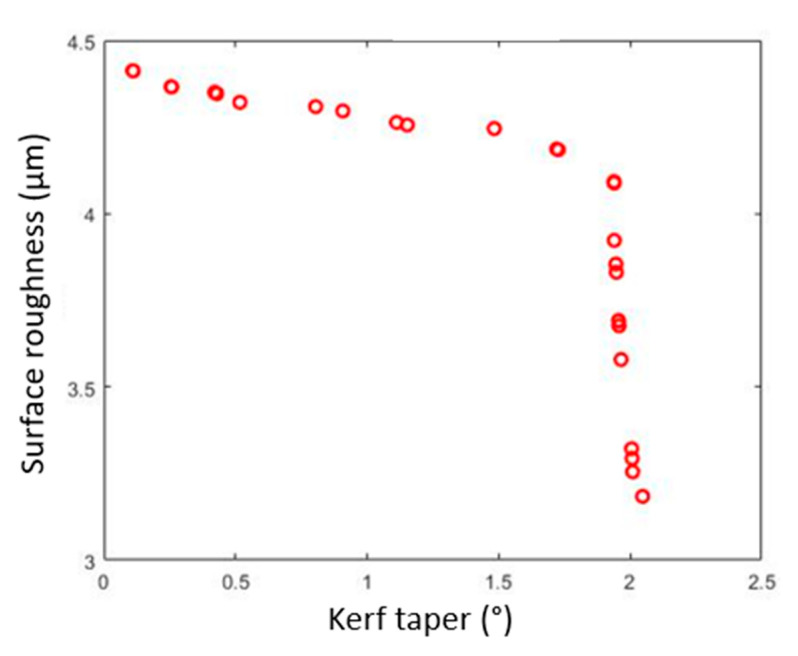
Sample pareto optimal front of SSO algorithm for optimized AWJC parameters.

**Figure 12:**
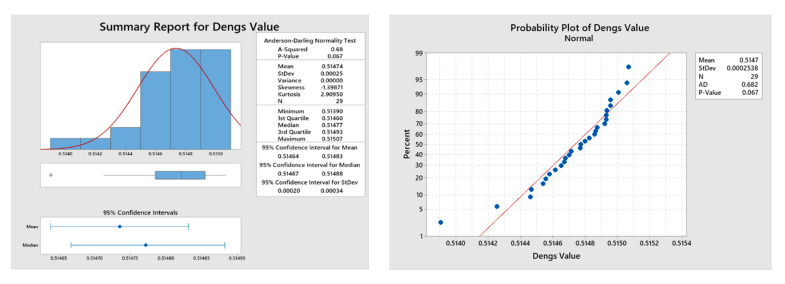

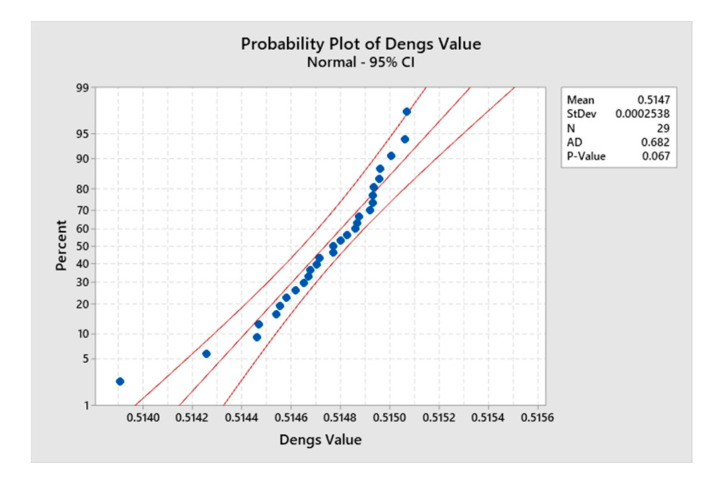
Statistical validation of results obtained from 29 runs.

The optimization was executed by using the SSO algorithm with the parameter settings of 30 number of salps with a maximum of 100 iterations. During the optimization, the parameters of SSO were self-tuned for obtaining global optimal solutions that satisfy the goal of objective functions. The algorithms were executed twenty-nine times to attain ingenious pareto-optimal settings. From each execution, a set of pareto-optimal solutions were attained and the best one among the optimal front was selected through Deng’s statistical approach [29]. The optimization performance of the SSO algorithm has been shown in the sample pareto-optimal front (Figure 11). Among the obtained optimal parameter settings through each run, the best one was attained by implementing the Deng’s statistical ranking approach. The solutions were ranked based on the Deng’s value, as mentioned in Table 9. From the table, a higher Deng’s value of 0.51507 was obtained at execution number 15 (As highlighted in bold) and their corresponding parameters, such as 1.004 wt% of r-GO, 600 mm/min of TS, 214 MPa of WP, and 4 mm of SOD, were global optimal parameter settings that provide an improved response characteristic value such as *Kt* of 2.067° and *Ra* of 3.18 µm, respectively. In addition, a statistical analysis (Anderson–Darling normality test) was performed to validate the obtained solutions at a 95% confidence level (Figure 12). The results of statistical analysis revealed that the obtained solutions are statistically significant and are normally scattered against actual values, which confirms the efficacy of the proposed optimization algorithm.

Confirmation experiments were conducted to assess the rationality of the proposed optimization approach. The confirmation experiments were repeated thrice with the obtained optimal AWJC parameters through the SSO algorithm and their average values are presented in Table 10. The table shows that the correlation between predicted and experimentally measured response values is relatively good, with an average error of 3.38% for *Kt*, and 3.77% for *Ra*, respectively. Therefore, the proposed SSO algorithm can be adequate for obtaining optimal processing conditions during AWJC of r-GO reinforced FILs for better cut quality characteristics.

## 5. Conclusions

In this study, a hybrid approach of a parametric tuned ANFIS-salp swarm optimization algorithm has been used for the modeling and optimization of AWJC parameters during the processing of novel fiber intermetallic laminates. Based on the results of the proposed hybrid approaches, the following inferences were attained:

- The Box–Behnken design-based response surface methodology approach has been effectively utilized for designing the experimental trails with a viable number of experiments to minimize the time and cost.
- The MFO algorithm was found to be outperformed in the training of the ANFIS network parameters with an average MAPE of 0.16838 for kerf taper and 0.10713 for surface roughness, and RMSE of 0.00895 for kerf taper and 0.01315 for surface roughness, respectively.
- The hybrid MFO-ANFIS approach was found to be an efficient approach for intelligent modeling of the AWJC process to correlate its process parameters and response characteristics, with average training and testing errors of 0.0056% and 0.0163% for kerf taper, and 0.0088% and 0.0231% for surface roughness, respectively.
- The optimal AWJC conditions for improved quality characteristics have been achieved through a metaheuristic salp swarm optimization algorithm, which are: 1.004 wt% of r-GO, 600 mm/min of TS, 214 MPa of WP, and 4 mm of SOD for an improved response characteristic value such as *Kt* of 2.067° and *Ra* of 3.18 µm, respectively.
- The results of confirmation studies revealed that the proposed optimization approach can be efficient in the prediction of optimal AWJC parameters with an error between the experimental and SSO algorithm predicted values of 3.38% for *Kt* and 3.77% for *Ra*, respectively.

## Figures and Tables

**Table 1:** Design matrix and experimentally measured response values.

Exp. Trial	Processing Parameters				Measured Responses	
	r-GO Addition (wt%)	Traverse Speed (mm/min)	Waterjet Pressure (MPa)	Stand-off Distance (mm)	Kerf Taper (°)	Surface Roughness (µm)
1	1	400	250	4	2.488	5.04
2	1	500	300	4	2.278	4.85
3	2	600	250	3	2.221	5.01
4	0	500	200	3	1.988	4.69
5	1	600	250	4	2.187	5.12
6	2	500	250	4	2.191	4.98
7	1	500	250	3	2.099	4.67
8	1	500	250	3	2.132	4.74
9	1	400	250	2	2.563	5.24
10	1	600	200	3	1.966	5.03
11	2	500	250	2	2.43	4.73
12	1	400	200	3	2.458	5.18
13	2	400	250	3	2.385	4.57
14	2	500	300	3	2.109	4.66
15	1	500	300	2	2.362	5.16
16	0	400	250	3	2.474	4.83
17	0	500	250	2	2.298	5.13
18	2	500	200	3	2.253	4.65
19	1	500	250	3	2.094	4.68
20	1	400	300	3	2.366	4.81
21	0	500	250	4	2.387	4.4
22	1	500	250	3	2.077	4.71
23	1	500	250	3	2.103	4.75
24	0	600	250	3	2.203	4.72
25	1	500	200	2	2.275	5.28
26	1	500	200	4	2.143	5.06
27	1	600	300	3	2.32	5.23
28	0	500	300	3	2.452	4.5
29	1	600	250	2	2.376	5.53

**Table 2:** Statistical analysis for *Kt*.

Source	SS	DOF	MS	F-Value	Prob > F
Model	0.699	14	0.049	111.7	<0.0001
wt% of r-GO	0.003	1	0.003	8.4475	0.0115
Traverse Speed	0.177	1	0.177	397.44	<0.0001
Waterjet Pressure	0.053	1	0.053	120.36	<0.0001
Stand-off Distance	0.03	1	0.03	73.901	<0.0001
Residual	0.006	14	0.006		
Lack of Fit	0.004	10	0.004	1.1723	0.4765
Pure Error	0.001	4	0.0003		
Cor Total	0.706	28			
R^2^	0.991	Adj. R^2^	0.982	Ade. Prec.	37.59

**Table 3:** Statistical analysis for *Ra*.

Source	SS	DOF	MS	F-Value	Prob > F
Model	2.022	14	0.144	130.1	<0.0001
wt% of r-GO	0.009	1	0.009	8.17	0.0126
Traverse Speed	0.078	1	0.078	70.63	<0.0001
Waterjet Pressure	0.038	1	0.038	34.71	<0.0001
Stand-off Distance	0.218	1	0.218	197.0	<0.0001
Residual	0.015	14	0.001		
Lack of Fit	0.010	10	0.001	0.84	0.6255
Pure Error	0.005	4	0.001		
Cor Total	2.038	28			
R^2^	0.992	Adj. R^2^	0.9847	Ade. Prec.	46.14

**Table 4:** FIS parameters used for the optimization.

Parameters	Representation	Values/Range
RADII − Cluster radius	Four input parameters and a response (either MRR or kerf taper or surface roughness)	0.13 to 0.5
Quash factor	To multiply RADII values	2 to 3
% of data for training FIS model	Total number of experiments	65% to 80%

**Table 5:** Parameters used in PSO and MFO algorithms for optimizing ANFIS parameters.

PSO Algorithm		MFO Algorithm	
Parameter	Value	Parameter	Value
Learning factors (*C1* & *C2*)	2 & 2	Position of moth close to the flame (*t*)	−1 to −2
Inertia weight (*ω*)	0.6	Update mechanism	Logarithmic spiral
Particle size (*N*)	30	No. of moths (*N*)	30
No. of iterations (*nitr*)	100	No. of iterations (*nitr*)	100

**Table 6:** Parameters of DFO algorithm for optimizing ANFIS parameters.

Parameters	Value/Equation
No. of dragonflies (nd)	100
No. of iterations (nitr)	100
Inertia weight (w) (w_max_ = 0.9 and w_min_ = 0.2)	$w=w_{max}-\left(w_{max}-w_{min}\right)\frac{itr}{nitr}$
Separation weight	$sw=0.1-\frac{0.1\;*\;itr}{nitr}$
Alignment weight	$aw=0.1-\frac{0.1\;*\;itr}{nitr}$
Cohesion weight	$cw=0.1-\frac{0.1\;*\;itr}{nitr}$
Food factor	ff=2 ∗ rand
Enemy factor	$ef=0.1-\frac{0.1\;*\;itr}{nitr}$
Achieve size	100

**Table 7:** Optimal ANFIS parameters.

Response	Algorithm	RADII Value					% Training Data	Quash Factor	T_Error_	C_Error_	RMSE	MAPE
		% r-GO	TS	WP	SOD	*Kt*/*Ra*						
*Kt* (°)	DFO	0.241	0.491	0.245	0.441	0.401	0.8	3	0.0056	0.0163	0.00897	0.16888
	MFO	0.473	0.309	0.383	0.352	0.191	0.8	2.87	0.0056	0.0163	0.00895	0.16838
	PSO	0.442	0.439	0.377	0.371	0.304	0.77	2.34	0.0056	0.0188	0.00991	0.20857
*Ra* (µm)	DFO	0.272	0.320	0.316	0.418	0.258	0.8	3	0.0088	0.0232	0.01317	0.10968
	MFO	0.278	0.5	0.435	0.448	0.260	0.79	2.10	0.0088	0.0231	0.01315	0.10713
	PSO	0.198	0.320	0.323	0.341	0.382	0.77	2.60	0.0088	0.0233	0.01323	0.11361

**Table 8:** Parameters of SSO algorithm.

Parameter	Value
C1-coefficient to control exploration and exploitation	$2e^{-{\left(\frac{4*it}{nitr}\right)}^{2}}$
C2 and C3	Random value between 0 and 1
No. of salps (*N*)	30
No. of iterations (*nitr*)	100

**Table 9:** Optimal AWJC parameters and their corresponding response values for 29 runs.

Run No.	r-GO (wt%)	TS (mm/min)	WP (MPa)	SOD (mm)	*Kt* (°)	*Ra* (µm)	Deng’s Value	Rank
1	1.140	600.00	209.98	4.0000	2.002	3.233	0.51446	27
2	1.094	600.00	210.25	4.0000	2.007	3.222	0.51454	25
3	1.178	600.00	213.33	4.0000	2.021	3.202	0.51470	18
4	1.089	600.00	213.43	4.0000	2.034	3.183	0.51486	12
5	1.038	600.00	208.35	3.9989	1.999	3.282	0.51425	28
6	1.024	600.00	213.83	4.0000	2.057	3.180	0.51501	3
7	1.011	600.00	212.76	4.0000	2.046	3.184	0.51493	8
8	1.016	600.00	210.29	4.0000	2.018	3.219	0.51462	22
9	1.097	600.00	213.71	4.0000	2.035	3.183	0.51487	11
10	1.015	600.00	212.83	4.0000	2.046	3.183	0.51493	7
11	1.078	600.00	213.41	4.0000	2.037	3.182	0.51488	10
12	1.138	599.99	211.82	4.0000	2.014	3.199	0.51467	20
13	1.159	600.00	211.95	4.0000	2.014	3.203	0.51465	21
14	1.131	599.99	209.94	4.0000	2.003	3.233	0.51447	26
**15**	**1.004**	**600.00**	**214.00**	**4.0000**	**2.067**	**3.180**	**0.51507**	**1**
16	1.195	598.37	212.03	4.0000	2.011	3.224	0.51455	24
17	1.017	600.00	214.29	4.0000	2.066	3.180	0.51506	2
18	1.051	600.00	217.75	3.9139	2.053	3.285	0.51458	23
19	1.070	600.00	212.08	4.0000	2.025	3.190	0.51477	15
20	1.060	600.00	213.80	4.0000	2.045	3.181	0.51493	6
21	1.099	600.00	212.83	3.9998	2.027	3.186	0.51480	14
22	1.000	600.00	211.67	4.0000	2.036	3.194	0.51483	13
23	1.044	600.00	213.77	4.0000	2.049	3.181	0.51496	4
24	1.047	600.00	213.81	4.0000	2.049	3.181	0.51496	5
25	1.088	600.00	212.28	4.0000	2.024	3.189	0.51477	16
26	1.137	600.00	211.91	4.0000	2.015	3.198	0.51468	19
27	1.068	600.00	211.49	4.0000	2.020	3.198	0.51471	17
28	1.176	599.92	207.06	3.9997	1.985	3.351	0.51390	29
29	1.034	600.00	213.09	4.0000	2.044	3.182	0.51492	9

**Table 10:** Results of validation experiments for AWJC parameters obtained through SSO algorithm.

Responses	SSO predicted	Experimental	Error %
Kerf taper (°)	2.067	2.137	3.38
Surface roughness (µm)	3.180	3.168	3.77
